# Cancer types with high numbers of driver events are largely preventable

**DOI:** 10.7717/peerj.12672

**Published:** 2022-01-05

**Authors:** Aleksey V. Belikov, Sergey V. Leonov

**Affiliations:** Laboratory of Innovative Medicine, School of Biological and Medical Physics, Moscow Institute of Physics and Technology, Dolgoprudny, Moscow Region, Russia

**Keywords:** Epidemiology, External risk, Modifiable risk, Population attributable fraction, Preventable cause

## Abstract

There is a long-standing debate on whether cancer is predominantly driven by extrinsic risk factors such as smoking, or by intrinsic processes such as errors in DNA replication. We have previously shown that the number of rate-limiting driver events per tumor can be estimated from the age distribution of cancer incidence using the gamma/Erlang probability distribution. Here, we show that this number strongly correlates with the proportion of cancer cases attributable to modifiable risk factors for all cancer types except the ones inducible by infection or ultraviolet radiation. The correlation was confirmed for three countries, three corresponding incidence databases and risk estimation studies, as well as for both sexes: USA, males (*r* = 0.80, *P* = 0.002), females (*r* = 0.81, *P* = 0.0003); England, males (*r* = 0.90, *P* < 0.0001), females (*r* = 0.67, *P* = 0.002); Australia, males (*r* = 0.90, *P* = 0.0004), females (*r* = 0.68, *P* = 0.01). Hence, this study suggests that the more driver events a cancer type requires, the more of its cases are due to preventable anthropogenic risk factors.

## Introduction

It is not known whether intrinsic or extrinsic factors play the key causative role in cancer. Historically, extrinsic factors such as smoking, carcinogenic chemicals and fumes, ionizing and ultraviolet radiation, were the most demonstrative risk factors for cancer (Doll, 1980; Fraumeni, 1982; Lyman, 1992). However, the role of intrinsic factors has been recently brought to attention by the work of Tomasetti and Vogelstein. They proposed that the majority of cancers develop due to replicative mutations occurring during the stem cell division (Tomasetti & Vogelstein, 2015; Tomasetti, Li & Vogelstein, 2017). As this challenges the widely accepted dominant role of extrinsic risk factors, further quantitative studies of the extrinsic versus intrinsic factors contribution to carcinogenesis are required (Wu et al., 2018).

We have previously shown that the number of rate-limiting driver events per tumor can be estimated from the age distribution of cancer incidence using the gamma/Erlang probability distribution, both for adult (Belikov, 2017) and childhood (Belikov, Vyatkin & Leonov, 2021) cancers. Here, we study the correlation of this number with the percentage of cancer cases due to modifiable risk factors. This is an often-used parameter in epidemiological studies, and is also called the population attributable fraction (PAF) (Mansournia & Altman, 2018). It shows, for example, what percentage of lung cancer cases are caused by smoking tobacco. Combined PAF shows the overall contribution of all potentially modifiable risk factors, which usually include air pollution, occupational hazards, ionizing radiation, smoking, alcohol, poor diet, insufficient exercise, obesity, infection and ultraviolet radiation. By definition, PAF is proportional to the prevalence of the exposure to the risk factor and the relative risk of cancer associated with such exposure (Mansournia & Altman, 2018). The relative risk magnitude, in turn, characterizes carcinogenic strength of the risk factor. Hence, we hypothesized that prevalent and strong risk factors should induce much more carcinogenic (driver) events in the general population than less prevalent and weak risk factors, as well as internal processes alone.

Indeed, we show that the numbers of driver events per tumor predicted by the gamma/Erlang distribution strongly correlate with combined PAFs for most cancers, with the exception of cancers with the large contribution from infection or ultraviolet radiation.

This suggests that cancer types with higher numbers of driver events are more dependent on anthropogenic risk factors.

## Methods

### Population attributable fractions data

Population attributable fractions (PAFs) combining all risk factors were obtained directly from published open-access articles separately for each cancer type and sex.

PAFs for USA were obtained from Table 2 in Islami et al. (2018). Briefly, Islami et al. applied a simulation method in which numbers from repeated draws were generated for all relative risks, exposure levels, and numbers of cancer cases and deaths, allowing for uncertainty in the data. The simulation process was replicated 1000 times for each sex and age-group stratum. The numbers from repeated draws were used to calculate the proportion and number of attributable cancer cases and deaths and their 95% confidence intervals. By using exposure prevalence (*Pi*) at the exposure category *i* and the corresponding relative risks (*RRi*), PAFs for categorical exposure variables for each stratum of sex and age group were calculated using the following formula:

\begin{eqnarray*}PAF= \frac{\sum {P}_{i} \left( R{R}_{i}-1 \right) }{\sum {P}_{i} \left( R{R}_{i}-1 \right) +1} \end{eqnarray*}

Islami et al. used the above approximate formula for all associations, with a few exceptions. All cervical cancers were attributed to human papillomavirus infection and all Kaposi sarcomas to Kaposi sarcoma herpesvirus/human herpesvirus 8 infection. Because of the lack of data on anal human papillomavirus infection, 88% of anal cancers were attributed to human papillomavirus 10 before applying the simulation method. PAFs for excess ultraviolet radiation-associated melanomas were estimated using the difference between observed melanoma incidence rates by sex and age group in the general population and the rates in blacks. To calculate the overall attributable proportion and number of cancer cases or deaths for a given cancer type when there were several risk factors, it was assumed that the risk factors had no interactions.

PAFs for England were obtained from Table 2 in Brown et al. (2018). Briefly, Brown et al. calculated PAFs for most risk factors using the same standard formula as Islami et al. PAFs for Epstein–Barr virus, human papillomavirus, Kaposi sarcoma herpesvirus/human herpesvirus 8, and diagnostic radiation were obtained by Brown et al. from other published studies. PAFs for all risk factors combined, for each cancer type and for all cancers combined, were obtained by first applying the first relevant PAF to the total number of observed cases, to obtain the number of cases attributable to that factor only. Each subsequent PAF was applied only to the number of observed cases not yet explained by the risk factors applied earlier. This aggregation method avoids overestimating PAFs for all risk factors combined but does not account for cases caused by exposure to risk factors in combination, *e.g.,* the synergistic effect.

PAFs for Australia were obtained from Table 2 in Whiteman et al. (2015a). Briefly, Whiteman et al. calculated PAFs using the same standard formula as Islami et al. and Brown et al. for all risk factors with the exception of some infections, smoking and ultaviolet radiation (Whiteman et al., 2015b). This method does not permit estimation of the fractions of cancers arising through synergistic effects of causal factors (Whiteman et al., 2015b). For Epstein–Barr virus and human papillomavirus, where mechanistic knowledge strongly suggests that the presence of infection in a cancer is sufficient to infer that infection caused the cancer, the PAF was assumed to be equivalent to the prevalence of viral DNA in tumour cells (Antonsson et al., 2015). Kaposi sarcoma herpesvirus is recognised as a necessary cause of Kaposi sarcoma, and thus the PAF was assumed to be 100% (Antonsson et al., 2015). The number of lung cancer cases expected in Australian adults in the absence of smoking was calculated by applying the estimated incidence rates of lung cancer in never smokers in the CPS II study to the population of Australia (Pandeya et al., 2015). The number and percentage of lung cancer cases attributable to smoking was then calculated by subtracting the expected number of cases from those actually observed (Pandeya et al., 2015). For the primary melanoma analysis, the difference was estimated between the observed numbers of melanoma cases in Australian residents (*i.e.,* ‘exposed’ to high ambient ultraviolet radiation in Australia) and the expected number of cases assuming the population was exposed to levels of ambient ultraviolet radiation experienced by an ‘ancestral’ population for many Australians - the UK population (Olsen et al., 2015).

No modification or processing of PAF data was performed.

### USA incidence data

United States Cancer Statistics Public Information Data: Incidence 1999–2012 was downloaded from the Centers for Disease Control and Prevention Wide-ranging OnLine Data for Epidemiologic Research (CDC WONDER) online database (http://wonder.cdc.gov/cancer-v2012.HTML). The United States Cancer Statistics (USCS) are the official federal statistics on cancer incidence from registries having high-quality data for 50 states and the District of Columbia. Data are provided by The Centers for Disease Control and Prevention National Program of Cancer Registries (NPCR) and The National Cancer Institute Surveillance, Epidemiology and End Results (SEER) program. Results were grouped by 5-year Age Groups and Crude Rates were selected as output. Crude Rates are calculated as the number of new cancer cases reported each calendar year per 100 000 population in each 5-year age group. The data were downloaded separately for males and females for each cancer type listed in Table 2 in the publication by Islami et al. (2018).

### England incidence data

England cancer incidence data were downloaded from the European Cancer Information System (ECIS) Data explorer (https://ecis.jrc.ec.europa.eu/explorer.php?0-11-UK2-2244-1,23-All6-5,845-1999,20127-2CRatesByCancerX0_10-ASR_EU_NEW). The ECIS database contains the aggregated output and the results computed from data submitted by population-based European cancer registries participating in Europe to the European Network of Cancer Registries – Joint Research Centre (ENCR-JRC) project on ”Cancer Incidence and Mortality in Europe”. Years of observation were limited to 1999–2012 period, to match the USA data. Incidence is calculated as the number of new cancer cases reported each calendar year per 100,000 population in each 5-year age group. The data were downloaded separately for males and females for each cancer type listed in Table 2 in the publication by Brown et al. (2018), except for vulva and vagina cancers, as their selection was not possible in ECIS Data explorer.

### Australia incidence data

Australia cancer incidence data were downloaded from the Cancer Incidence in Five Continents (CI5) Volume XI Age-specific curves Online Analysis tool (http://ci5.iarc.fr/CI5-XI/Pages/age-specific-curves_sel.aspx). CI5 is published approximately every five years by the International Agency for Research on Cancer (IARC) and the International Association of Cancer Registries (IACR) and provides comparable high quality statistics on the incidence of cancer from cancer registries around the world. Volume XI contains information from 343 cancer registries in 65 countries for cancers diagnosed from 2008 to 2012. Incidence is calculated as the number of new cancer cases reported each calendar year per 100 000 population in each 5-year age group. The data were downloaded separately for males and females for each cancer type listed in Table 2 in the publication by Whiteman et al. (2015a).

### Estimation of the number of driver events per tumor

For analysis, the incidence data were imported into GraphPad Prism 9 (http://www.graphpad.com/). The following age groups were selected: “5–9 years”, “10–14 years”, “15–19 years”, “20–24 years”, “25–29 years”, “30–34 years”, “35–39 years”, “40–44 years”, “45–49 years”, “50–54 years”, “55–59 years”, “60–64 years “, “65–69 years”, “70–74 years”, “75–79 years” and “80–84 years”. Prior age groups were excluded due to possible contamination by childhood subtype incidence, and “85+ years” was excluded due to an undefined age interval. If in the first several age groups (“5–9 years”, “10–14 years”, “15–19 years”) incidence initially decreased with age, reflecting contamination by childhood subtype incidence, these values were removed until a steady increase in incidence was detected. The middle age of each age group was used for the x values, *e.g.,* 17.5 for the “15–19 years” age group. Incidence (new cancer cases per calendar year per 100,000 population) for each age group and each cancer type was used for the y values. Data for different countries, as well as for males and females, were analyzed separately. Data were analyzed with Nonlinear regression using the following User-defined equation for the gamma distribution:

\begin{eqnarray*}Y=A\ast (x\wedge (k-1))\ast (\text{exp}(-x/b))/((b\wedge k)\ast \text{gamma}(k)) \end{eqnarray*}

The amplitude parameter *A* was constrained to “Must be between zero and 100000.0” and scale and shape parameters *b* and *k* to “Must be greater than 0.0”. “Initial values, to be fit” for all parameters were set to 50. All other settings were kept at default values, *e.g.,* Least squares fit and No weighting.

The numerical value of the shape parameter *k* rounded to the nearest integer was interpreted as the number of driver events per tumor (Belikov, 2017).

### Correlation of the predicted numbers of driver events per tumor with PAFs

Obtained *k* values were correlated to population attributable fractions (PAFs) in GraphPad Prism 6 using the inbuilt Correlation tool at default settings, *e.g.,* Pearson correlation with two-tailed *P* value. Cancer types were sorted into two classes, and correlation was performed separately for each class. Cancer types in which infection (*Helicobacter pylori*, hepatitis B virus, hepatitis C virus, Kaposi sarcoma herpesvirus/ human herpesvirus 8, human immunodeficiency virus and human papillomavirus) or ultraviolet radiation contributed to more than 30% of cases, for a given country according to the published PAF data (Whiteman et al., 2015a; Brown et al., 2018; Islami et al., 2018), were assigned to Class 2 (non-anthropogenic). The rest were assigned to Class 1 (anthropogenic), which included cancers with substantial contribution from air pollution, occupational exposure, exposure to ionizing radiation, smoking and exposure to secondhand smoke, alcohol intake, poor diet (red and processed meat, insufficient fiber, vegetables, fruit and calcium), excess body weight, insufficient physical activity, insufficient breastfeeding, postmenopausal hormone therapy and oral contraceptives, according to the published PAF data (Whiteman et al., 2015a; Brown et al., 2018; Islami et al., 2018).

## Results

**Figure 1:**
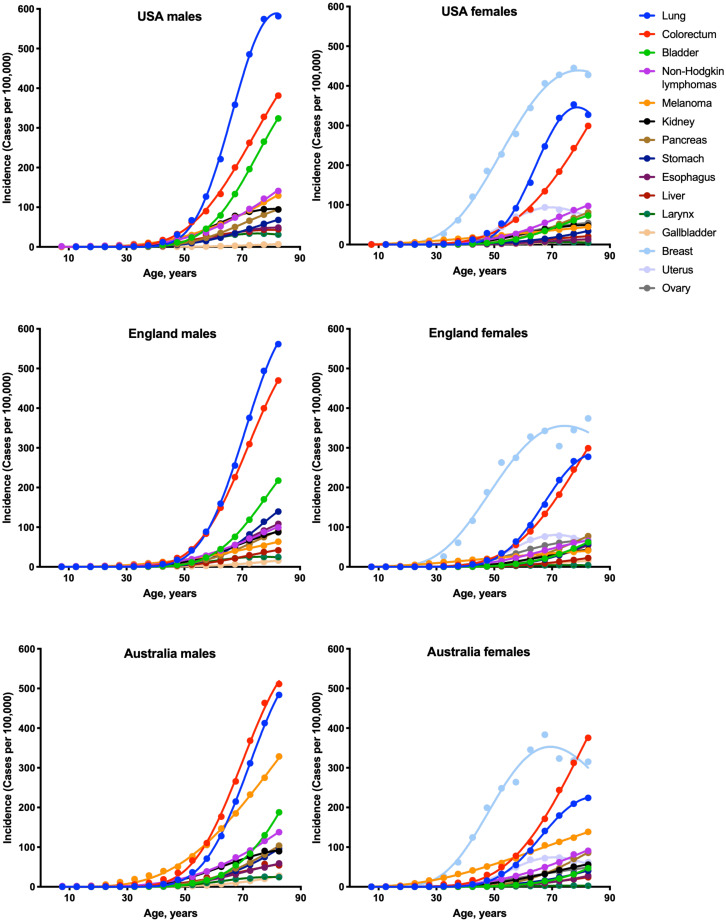
Fits of the gamma distribution to the actual incidence data for various cancers. Only the cancer types with data available for all three countries are shown. Cancer types with very low incidence are not shown.

To estimate the numbers of driver events per tumor, the gamma distribution was fitted to the actual age distributions of incidence separately for males and females in three countries: USA, England and Australia (Fig. 1 and Table 1). The fits were generally excellent (*R*^2^ = 0.99 for 22 cancer types), except for brain cancer (*R*^2^ = 0.98), thyroid cancer (*R*^2^ = 0.97), and several virus-induced cancers: pharyngeal (*R*^2^ = 0.98), nasopharyngeal (*R*^2^ = 0.93), vulvar (*R*^2^ = 0.98), cervical (*R*^2^ = 0.77), Kaposi sarcoma (*R*^2^ = 0.67) and Hodgkin lymphoma (*R*^2^ = 0.34). Due to the unsatisfactory fits, the last three cancer types were excluded from the further analysis. Successful fitting of the remaining 27 cancer types allowed the estimation of the numbers of driver events per tumor using the shape parameter of the gamma distribution.

**Table 1:** Goodness of fit (R^2^) of the gamma distribution to the actual cancer incidence data.

Cancer type	England		USA		Australia	
	Males	Females	Males	Females	Males	Females
Lung	0.9996	0.9989	0.9990	0.9973	0.9997	0.9994
Uterus	–	0.9965	–	0.9954	–	0.9934
Mesothelioma	0.9997	0.9980	ND	ND	ND	ND
Larynx	0.9989	0.9964	0.9997	0.9945	0.9965	0.9838
Pancreas	0.9999	0.9999	1.000	0.9998	0.9995	0.9982
Bladder	0.9998	0.9998	0.9997	0.9996	0.9998	0.9994
Myeloma	0.9999	0.9998	0.9994	0.9992	ND	ND
Kidney	0.9991	0.9986	0.9985	0.9959	0.9970	0.9985
Gallbladder	0.9998	0.9991	0.9994	0.9996	0.9996	0.9992
Ovary	–	0.9990	–	0.9990	–	0.9956
Colorectum	0.9998	0.9997	0.9993	0.9987	0.9987	0.9987
Liver^*^	0.9987	0.9994	0.9839	0.9985	0.9797	0.9975
Esophagus	0.9997	0.9997	0.9999	0.9991	0.9989	0.9981
Non-Hodgkin lymphoma	0.9970	0.9976	0.9973	0.9969	0.9985	0.9974
Oral cavity	0.9912	0.9973	ND	ND	ND	ND
Oral cavity and pharynx	ND	ND	0.9971	0.9987	0.9972	0.9966
Breast	–	0.9828	–	0.9978	–	0.9887
Brain	0.9797	0.9775	ND	ND	ND	ND
Leukemia	0.9957	0.9961	ND	ND	0.9950	0.9912
Myeloid leukemia	ND	ND	0.9930	0.9891	ND	ND
Thyroid	0.9859	0.9251	0.9808	0.9870	ND	ND
Melanoma	0.9970	0.9962	0.9987	0.9945	0.9983	0.9984
Stomach	0.9988	0.9983	0.9993	0.9979	0.9999	0.9965
Anus^*^	0.9982	0.9934	0.9927	0.9827	0.9926	0.9700
Pharynx^*^	0.9848	0.9790	ND	ND	ND	ND
Nasopharynx^*^	0.9776	0.8836	ND	ND	ND	ND
Kaposi sarcoma^*^	0.6761	0.3861	0.1997	0.9906	0.7562	0.9942
Hodgkin lymphoma^*^	0.5301	0.1664	0.5702	0.1730	0.4513	0.1200
Penis^*^	0.9961	–	0.9982	–	0.9762	–
Cervix^*^	–	0.6003	–	0.9033	–	0.8016
Vagina^*^	–	ND	–	0.9985	–	0.9918
Vulva^*^	–	ND	–	0.9900	–	0.9773

**Notes.**

ND no incidence data in the database or no corresponding PAF data in the source publication

Asterisk (*) denotes cancers in which a viral infection contributes to more than 30% of cases, according to the published PAF data (Whiteman et al., 2015a; Brown et al., 2018; Islami et al., 2018).

Plotting the correlation of the number of driver events per tumor predicted from the gamma distribution with the estimated percentage of cases due to modifiable risk factors obtained from the published studies revealed that cancers appear to cluster into two classes. Class 1, which included the majority of cancers (18), demonstrated the linear correlation, whereas Class 2, representing the minority (9), clustered in the upper left corner of the plot in a cloud-like fashion. Investigation of the Class 2 revealed that it consists entirely of cancers with substantial (>30%) contribution of infection to their pathogenesis, plus the melanoma cancer. Class 2 was therefore named “non-anthropogenic”, as infections and ultraviolet radiation existed long before the advent of human civilization. Interestingly, all cancers in Class 1 were induced by factors that arose with human civilization, such as air pollution, occupational hazards, ionizing radiation, smoking, alcohol, poor diet, insufficient exercise, obesity, insufficient breastfeeding, postmenopausal hormone therapy and oral contraceptives. Therefore, Class 1 was termed “anthropogenic”.

**Figure 2:**
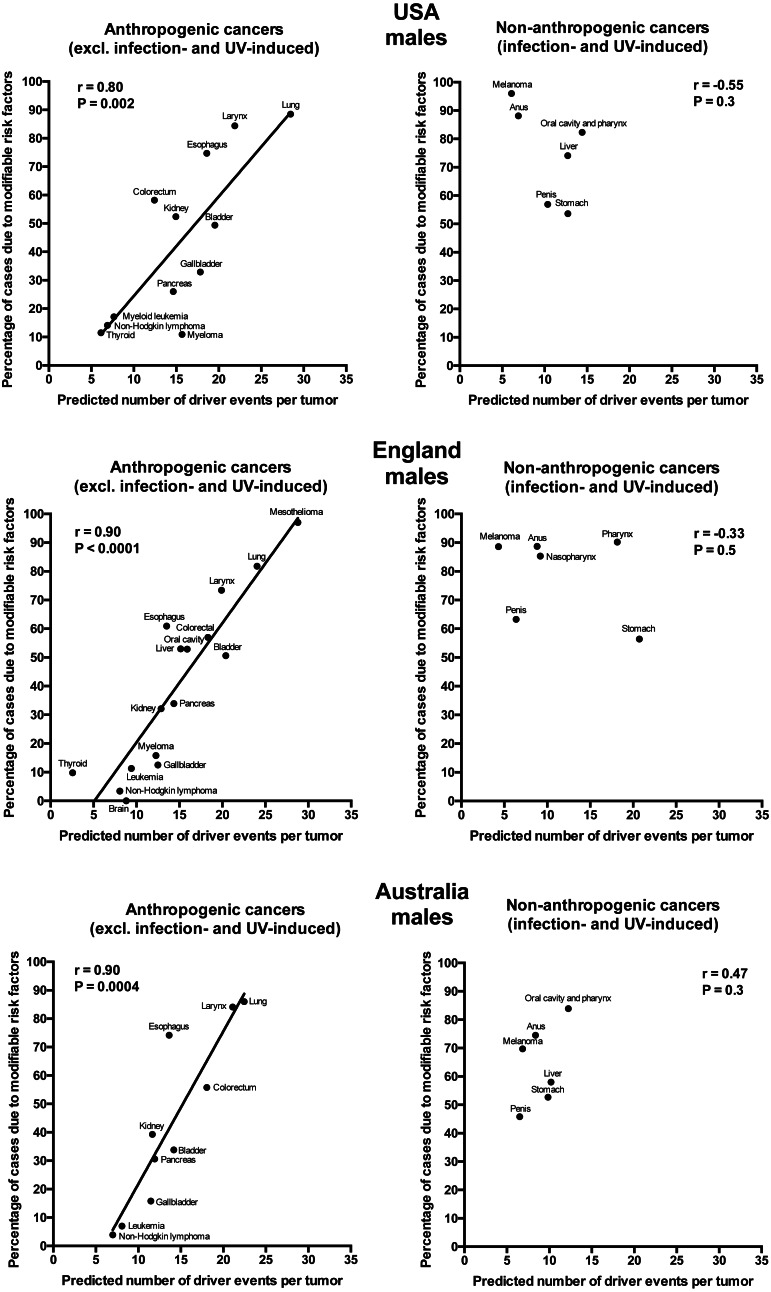
Correlation of the predicted numbers of driver events per tumor with the estimated percentages of cases due to modifiable risk factors for cancers in males.

**Figure 3:**
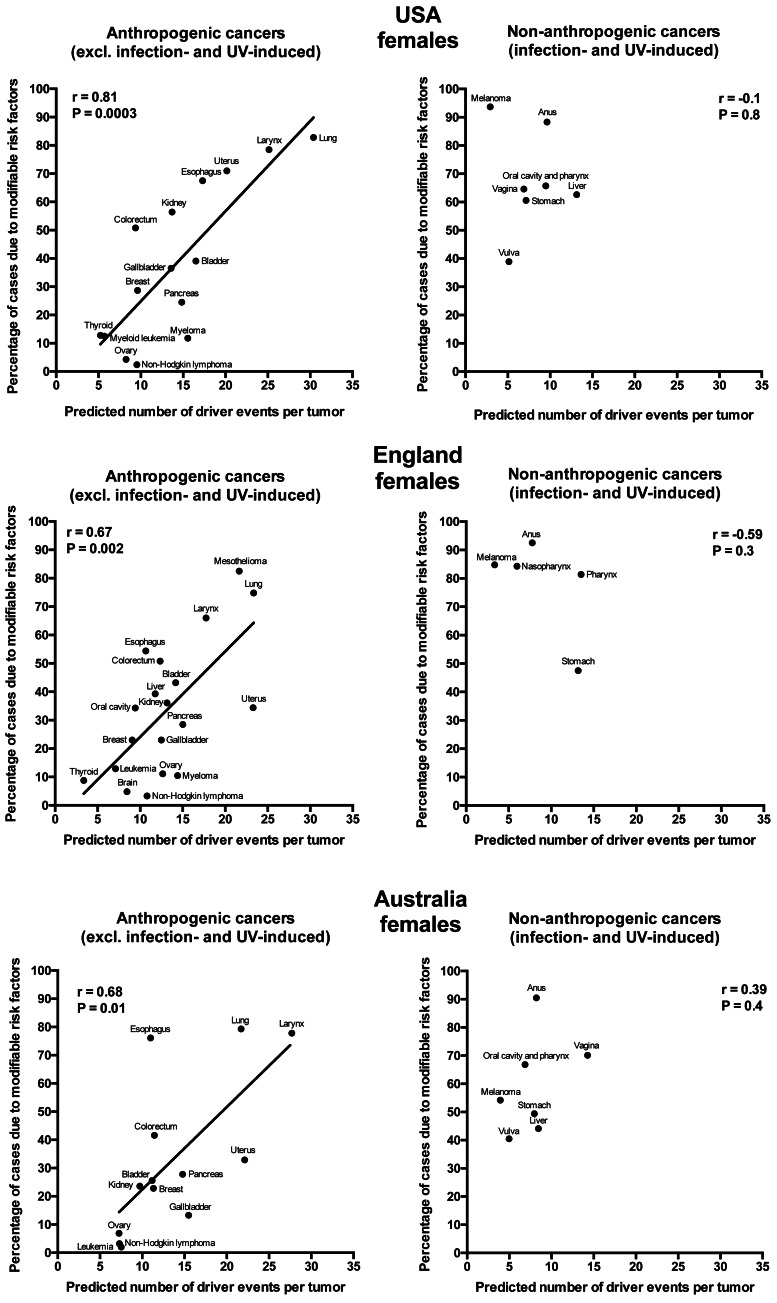
Correlation of the predicted numbers of driver events per tumor with the estimated percentages of cases due to modifiable risk factors for cancers in females.

**Figure 4:**
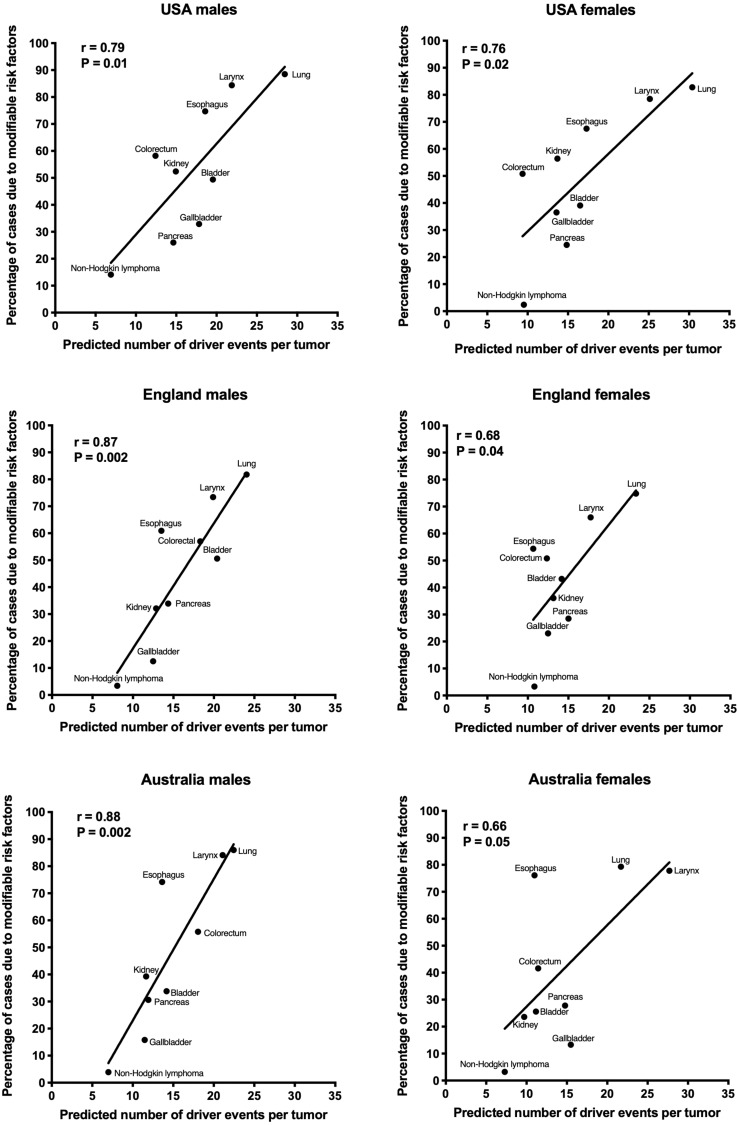
Correlation of the predicted numbers of driver events per tumor with the estimated percentages of cases due to anthropogenic risk factors. Only the cancer types with data available for all three countries and both sexes are shown.

The correlation of the predicted number of driver events per tumor with the estimated percentage of cases due to modifiable risk factors for cancers in males is shown in Fig. 2 and Table 2, and in females in Fig. 3 and Table 3. It can be seen that anthropogenic cancers indeed exhibit the strong significant positive correlation for all studied countries and for both sexes, whereas for non-anthropogenic cancers correlations are not significant. Amongst anthropogenic cancers, the correlation is stronger for males than for females. Interestingly, the correlation is stronger for USA females (*r* = 0.81, *P* = 0.0003) than for English (*r* = 0.67, *P* = 0.002) and Australian (*r* = 0.68, *P* = 0.01) females, but weaker for USA males (*r* = 0.80, *P* = 0.002) than for English (*r* = 0.90, *P* < 0.0001) and Australian (*r* = 0.90, *P* = 0.0004) males. This observation holds true even when identical sets of cancer types are compared (Fig. 4). These differences are likely explained by differing exposures to risk factors between countries and between sexes, as well as by variations in the screening, diagnostics and reporting protocols of different countries, and in the methodologies of those studies. The role of population genetics also cannot be ruled out.

**Table 2:** Predicted numbers of driver events per tumor and estimated percentages of cases due to anthropogenic risk factors for cancers in males.

Cancer type	England		USA		Australia	
	Predicted number of driver events per tumor	Estimated percentage of cases due to modifiable risk factors (Brown et al., 2018)	Predicted number of driver events per tumor	Estimated percentage of cases due to modifiable risk factors (Islami et al., 2018)	Predicted number of driver events per tumor	Estimated percentage of cases due to modifiable risk factors (Whiteman et al., 2015a)
Mesothelioma	29	97	–	ND	–	ND
Lung	24	82	28	89	22	86
Larynx	20	73	22	84	21	84
Bladder	20	51	20	49	14	34
Colorectum	18	57	12	58	18	56
Oral cavity	16	53	–	ND	–	ND
Liver	15	53	–	NA	–	NA
Esophagus	14	61	19	75	14	74
Pancreas	14	34	15	26	12	31
Kidney	13	32	15	52	12	39
Myeloma	12	16	16	11	–	ND
Gallbladder	12	13	18	33	11	16
Brain	9	0	–	ND	–	ND
Leukemia	9	11	–	ND	8	7
Myeloid leukemia	–	ND	8	17	–	ND
Non-Hodgkin lymphoma	8	3	7	14	7	4
Thyroid	3	10	6	12	–	ND

**Notes.**

ND no data in the source publication NA assigned to the non-anthropogenic group due to the strong contribution of a viral infection

**Table 3:** Predicted numbers of driver events per tumor and estimated percentages of cases due to anthropogenic risk factors for cancers in females.

Cancer type	England		USA		Australia	
	Predicted number of driver events per tumor	Estimated percentage of cases due to modifiable risk factors (Brown et al., 2018)	Predicted number of driver events per tumor	Estimated percentage of cases due to modifiable risk factors (Islami et al., 2018)	Predicted number of driver events per tumor	Estimated percentage of cases due to modifiable risk factors (Whiteman et al., 2015a)
Lung	23	75	30	83	22	79
Uterus	23	34	20	71	22	33
Mesothelioma	22	83	–	ND	–	ND
Larynx	18	66	25	79	28	78
Pancreas	15	29	15	25	15	28
Bladder	14	43	17	39	11	26
Myeloma	14	11	16	12	–	ND
Kidney	13	36	14	56	10	24
Gallbladder	12	23	14	37	15	13
Ovary	13	11	8	4	7	7
Colorectum	12	51	9	51	11	42
Liver	12	39	–	NA	–	NA
Esophagus	11	54	17	68	11	76
Non-Hodgkin lymphoma	11	3	10	2	7	3
Oral cavity	9	34	–	ND	–	ND
Breast	9	23	10	29	11	23
Brain	8	5	–	ND	–	ND
Leukemia	7	13	–	ND	8	2
Myeloid leukemia	–	ND	6	13	–	ND
Thyroid	3	9	5	13	–	ND

**Notes.**

ND no data in the source publication NA assigned to the non-anthropogenic group due to the strong contribution of a viral infection

## Discussion

One of the most interesting findings of this study is the clustering of all cancers into two classes, termed here anthropogenic and non-anthropogenic. The possible explanation for this dichotomy is that the human body managed to evolve some protective countermeasures against cancer risk factors that were present for millions of years, whereas it appears unprepared for the novel risk factors brought by our civilization. For example, ultraviolet radiation has been present on Earth since the beginning, and although melanocytes cannot completely protect their DNA, and a lot of DNA damage occurs, it is likely that they developed a very slow division rate (Halaban et al., 1986) to avoid conversion of this damage into mutations for as long as possible. This may explain why only few rate-limiting driver events are predicted for melanoma despite lots of DNA damage that melanocytes receive: rate-limiting in this case is cell division and not the DNA damage. Similarly, the human body had plenty of time to adapt to viruses and develop protective mechanisms, such as RNAi-mediated destruction of double-stranded RNA (Maillard et al., 2019), interferon-driven innate immune system (Schlee & Hartmann, 2016), and T- and B-cell responses of the adaptive immunity (Mueller & Rouse, 2008). This may explain why the incidence rates of virus-induced cancers are low, and less driver events are predicted than would be expected from the linear correlation. It is also clear that viruses are inducing cancer via different mechanisms than chemical carcinogens (Butel, 2000; Mesri, Feitelson & Munger, 2014), and thus the development of such cancers may not be described by the Poisson process underlying the Erlang distribution (Belikov, 2017; Belikov, Vyatkin & Leonov, 2021). Indeed, many of the virus-induced cancers have rather poor fits of the Erlang distribution to their age distributions of incidence (Table 1).

The strong positive correlation of the predicted number of driver events per tumor with the contribution from anthropogenic risk factors suggests that the higher is the number of driver events that are required for a given cancer type to appear, the less likely is for them to occur by chance (*e.g.,* due to replication errors), and the more dependent are they on anthropogenic carcinogens to be induced. This is in accord with the mainstream view that the environment and lifestyle are the major contributors to carcinogenesis, but conflicts with the recently proposed view that the majority of cancers develop due to replicative mutations occurring during stem cell division (Tomasetti & Vogelstein, 2015; Tomasetti, Li & Vogelstein, 2017). The latter view is based on predominantly mouse data handpicked from varied publications and processed through calculations with unobvious assumptions, and thus has been widely criticized (Ashford et al., 2015; Gotay, Dummer & Spinelli, 2015; O’Callaghan, 2015; Rozhok, Wahl & De Gregori, 2015; Giovannucci, 2016; Wu et al., 2016; Wensink, Vaupel & Christensen, 2017).

It is also interesting to speculate why the observed correlations are stronger for males than for females. One likely explanation is that males generally are more exposed to chemical mutagens, *e.g.,* during smoking and at dangerous industries (Whiteman et al., 2015a; Brown et al., 2018; Islami et al., 2018), directly inducing mutations in the DNA, some of which happen to be drivers. On the other hand, females have a higher contribution to cancer risk from disturbances in physiology, usually related to hormone levels, such as being obese, using oral contraceptives, undergoing postmenopausal hormone therapy or abstaining from breastfeeding (Whiteman et al., 2015a; Brown et al., 2018; Islami et al., 2018). These risk factors may not lead to an increase in the number of overall and driver mutations (discrete events), but promote cancer via changes in intracellular signaling levels or the microenvironment (gradual change) (Treviño, Wang & Walker, 2015; McNamara et al., 2016; Tahergorabi et al., 2016; Carroll, 2016; Quail & Dannenberg, 2019). The latter cannot be detected and counted using the gamma/Erlang distribution, which is capable of recognizing only discrete random events (Belikov, 2017; Belikov, Vyatkin & Leonov, 2021).

## Conclusions

Overall, this study shows that cancer types with high numbers of driver events are induced predominantly by anthropogenic carcinogens and lifestyle, and not by internal processes such as DNA replication. It implies that most carcinogenic alterations in those cancer types can be prevented by changing the lifestyle and environment.

## Supplemental Information

10.7717/peerj.12672/supp-1 Supplemental Information 1 Raw data and calculations

## Additional Information and Declarations

### Data Availability

The following information was supplied regarding data availability: The raw and processed data available in the Supplemental File.
